# Identification and quantification of plasma calciprotein particles with distinct physical properties in patients with chronic kidney disease

**DOI:** 10.1038/s41598-018-19677-4

**Published:** 2018-01-19

**Authors:** Yutaka Miura, Yoshitaka Iwazu, Kazuhiro Shiizaki, Tetsu Akimoto, Kazuhiko Kotani, Masahiko Kurabayashi, Hiroshi Kurosu, Makoto Kuro-o

**Affiliations:** 1 Division of Anti-Ageing Medicine, Center for Molecular Medicine, Jichi Medical University, Shimotsuke, Tochigi, Japan; 2 Department of Clinical Laboratory Medicine, Jichi Medical University, Shimotsuke, Tochigi, Japan; 3 Division of Nephrology, Department of Internal Medicine, Jichi Medical University, Shimotsuke, Tochigi, Japan; 4 Division of Community and Family Medicine, Center for Community Medicine, Jichi Medical University, Shimotsuke, Tochigi, Japan; 5 Department of Cardiovascular Medicine, Gunma University Graduate School of Medicine, Maebashi, Gunma, Japan; 6 AMED-CREST, Japan Agency for Medical Research and Development, Tokyo, Japan

## Abstract

Calciprotein particles (CPP) are solid-phase calcium-phosphate bound to serum protein fetuin-A and dispersed as colloids in the blood. Recent clinical studies indicated that serum CPP levels were increased with decline of renal function and associated with inflammation and vascular calcification. However, CPP assays used in these studies measured only a part of CPP over a certain particle size and density. Here we show that such CPP are mostly artifacts generated during processing of serum samples *in vitro*. The native CPP in fresh plasma are smaller in size and lower in density than those artifactual CPP, composed of fetuin-A carrying amorphous and/or crystalline calcium-phosphate, and increased primarily with serum phosphate levels. We have identified several physicochemical factors that promote aggregation/dissolution of CPP and transition of the calcium-phosphate from the amorphous phase to the crystalline phase *in vitro*, including addition of anti-coagulants, composition of buffer for sample dilution, the number of freeze-thaw cycles, the speed for sample freezing, and how many hours the samples were left at what temperature. Therefore, it is of critical importance to standardize these factors during sample preparation in clinical studies on CPP and to investigate the biological activity of the native CPP.

## Introduction

Animals with the bone made of calcium-phosphate have evolved mechanisms that prevent unwanted growth of calcium-phosphate crystals in extaosseous tissues. One of such mechanisms has been ascribed to serum protein fetuin-A^1^. Fetuin-A has the ability to bind to calcium-phosphate precipitates, thereby preventing them from growing into large crystals in the extracellular space. As a result, aggregates of fetuin-A molecules laden with tiny calcium-phosphate precipitates are generated to form into nanoparticles. These mineral-protein complexes, termed calciprotein particles (CPP), are dispersed as colloids^2^.

CPP can be synthesized *in vitro* by adding calcium and phosphate to serum-containing buffer followed by incubation at 37 °C for a few hours or days^3,4^. Under these conditions, two types of CPP, termed primary CPP and secondary CPP, are generated with different size, composition, and morphology. Primary CPP appear within a few hours after addition of calcium and phosphate, which are round particles with a hydrodynamic radius around 75 nm containing amorphous calcium-phosphate. After prolonged incubation, primary CPP spontaneously undergo transformation into secondary CPP, which are oval particles with a hydrodynamic radius around 120 nm containing crystalline calcium-phosphate. Both primary and secondary CPP can be precipitated by centrifugation at 16,000~24,000 g for 2 hours, however, the biological activity appears different. Secondary CPP have the ability to induce calcification in vascular smooth muscle cells and innate immune responses in macrophages in culture, but primary CPP are incapable of inducing smooth muscle cell calcification^5,6^.

Several clinical studies indicated that CPP were detectable in serum samples from patients with chronic kidney disease (CKD)^7,8^. In these studies, serum CPP levels were quantified as follows: First, the serum fetuin-A level (=F1) was measured using a human fetuin-A ELISA kit. Second, the serum was centrifuged at 16,000~24,000 g for 2 hours to precipitate CPP. Third, the fetuin-A level in the supernatant (=F2) was measured using the ELISA kit. Lastly, the fetuin-A reduction rate defined as (F1 − F2)/F1 was calculated and used as a surrogate for the serum CPP level. Using this “fetuin-A method”, these studies demonstrated that serum CPP levels were increased with decline of renal function and correlated with coronary artery calcification score, aortic pulse wave velocity, and serum markers for inflammation. The fact that CPP have the ability to induce smooth muscle cell calcification and innate immune responses *in vitro* have raised the possibility that CPP may be a causative agent for arteriosclerosis and chronic non-infectious inflammation that exacerbate prognosis of CKD patients.

The fetuin-A method has two major limitations. First, the lower the difference between F1 and F2, the lower the precision. Because the coefficient of variation (CV) of the human fetuin-A ELISA kits used in these studies is 2~5%, the fetuin-A reduction rate around this range or lower is considered unreliable. Second, the fact that the assay depends on validated human fetuin-A ELISA kits limits its direct application to experimental animals. To overcome these limitations, a new CPP assay was reported recently^9^. Taking advantage of the ability of bisphosphonates to bind specifically to calcium-phosphate crystals, Smith and his colleagues labeled CPP in serum with an infrared fluorescent bisphosphonate (OsteoSense) and then analyzed with flow cytometry. Unlike the fetuin-A method, this “flow cytometric method” can be directly applicable to experimental animals, count the number of CPP, and distinguish membrane-bound mineral particles such as exosomes from membrane-free CPP by using another fluorescent probe for cell membrane. However, the flow cytometric method can detect CPP larger than 100 nm in diameter, which is the principled limit of flow cytometry.

Here we show the development of another CPP assay using OsteoSense. After serum or plasma samples are inoculated with OsteoSense, CPP-bound OsteoSense is separated from unbound OsteoSense using a gel filtration spin column. The fluorescence intensity of the flow-through, which contains CPP, is quantified using an infrared fluorescence scanner and designated as the total CPP level. This “gel filtration method” is not only more sensitive, rapid, and less expensive than the fetuin-A method, but also directly applicable to experimental animals like the flow cytometric method. More importantly, the gel filtration method has revealed the presence of CPP that are smaller in size and lower in density than those measured by the fetuin-A method and the flow cytometric method. We also show that these low density CPP (L-CPP) actually represent the major form of CPP *in vivo*.

## Results

### A novel CPP assay

**Figure 1:**
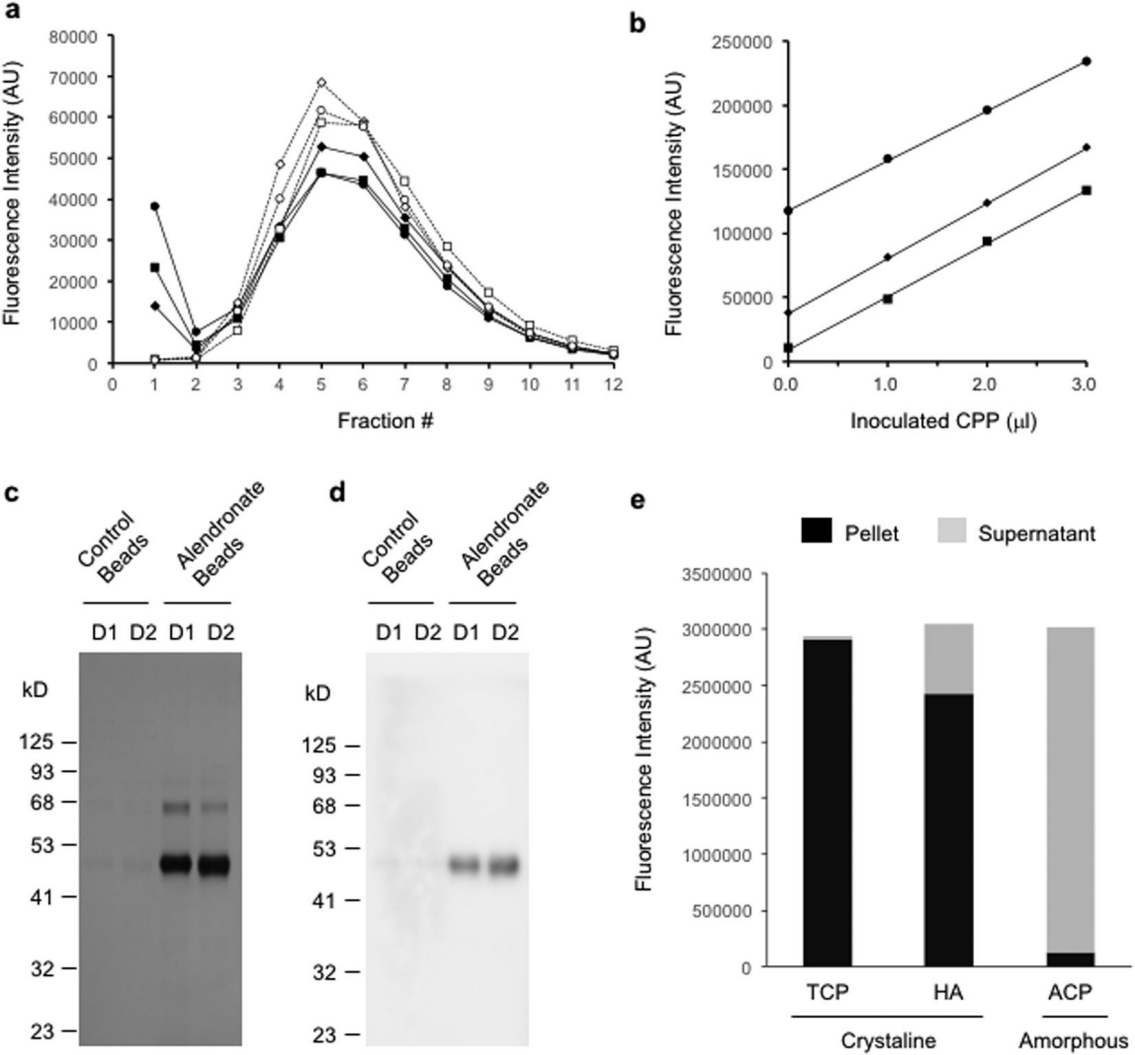
Bisphosphonate binds selectively and quantitatively to fetuin-A carrying crystalline calcium-phosphate in plasma. (**a**) Gel filtration chromatograms of plasma samples from three dialysis patients inoculated with OsteoSense in the absence (closed markers with solid lines) or presence (open markers with dot lines) of EDTA. (**b**) Linear relation between the amount of exogenous CPP and the OsteoSense fluorescence of the void fraction in the gel filtration method. Plasma samples from three dialysis patients were inoculated with known amounts of synthesized CPP. The total CPP levels were determined by the gel filtration method and plotted against the amount of exogenous CPP. (**c**) Pull-down of plasma CPP using bisphosphonate. Magnetic beads conjugated with or without alendronate (Alendronate Beads or Control Beads, respectively) were added to plasma samples from two dialysis patents (D1 and D2). The beads were washed with DMEM and then eluted with EDTA. The eluates were subjected to SDS-PAGE followed by silver staining. (**d**) As in (**c**), except that the eluates were subjected to immunoblot analysis using an antibody against human fetuin-A. (**e**) Binding of OsteoSense to amorphous or crystalline calcium-phosphate nanoparticles. Suspensions of calcium-phosphate nanoparticles were inoculated with OsteoSense and then centrifuged to precipitate the nanoparticles. OsteoSense in the supernatant was measured (grey bars) and subtracted from the total OsteoSense inoculated to calculate OsteoSense in the pellet (black bars). TCP, tricalcium phosphate; HA, hydroxyapatite; ACP, amorphous calcium-phosphate. The full-length gels/blots for (**c**) and (**d**) were shown in Supplementary information.

OsteoSense, bisphosphonate conjugated with an infared fluorescent dye, was added to serum samples from dialysis patients and incubated at 25 °C for 60 minutes. The samples were then applied to gel filtration spin columns (molecular weight cut-off at 40 kDa) and eluted stepwise with buffer containing physiological levels of calcium and phosphate (Dulbecco’s Modified Eagle Medium, DMEM). Two peaks were observed in chromatograms of OsteoSense fluorescence, a sharp peak at the high molecular weight (HMW) fraction (the void fraction) and a broad peak at low molecular weight (LMW) fractions (Fig. 1a). The HMW peak and the LMW peak represented CPP-bound OsteoSense and free OsteoSense, respectively, because dissolution of CPP by addition of EDTA to the samples resulted in disappearance of the HMW peak and reciprocal increase in the LMW peak (Fig. 1a). The HMW peak after the EDTA treatment was less than 1% of that before the EDTA treatment, indicating that the background fluorescence was negligible.

To verify that the fluorescence intensity of the void fraction was correlated linearly with the amount of CPP, we inoculated known amounts of synthesized CPP into serum samples and observed linear relation between the fluorescence intensity and the amount of inoculated exogenous CPP. The regression lines obtained by using different serum samples showed parallel slopes with different y-intercepts that represented the endogenous CPP levels (Fig. 1b). These findings verify that CPP can be quantified as an arbitrary unit of OsteoSense fluorescence intensity in the void fraction. The coefficient of variation (CV) was 2.2% in this “gel filtration method”.

To confirm the binding specificity of bisphosphonate to CPP, we generated magnetic beads surface-immobilized with alendronate and asked if the beads would pull down CPP specifically from plasma. The alendronate beads or the control beads (magnetic beads without alendronate conjugation) were added to plasma samples from dialysis patients. After washing the beads with DMEM, we treated them with EDTA to dissolve calcium-phosphate and subjected the eluates to SDS-PAGE. A robust band at ~50 kDa and a faint band at ~65 kDa were observed in the eluate from the alendronate beads (Fig. 1c). The ~65 kDa protein was presumably albumin, which was reported as one of the minor components of CPP^7^. The ~50 kDa protein was confirmed as human fetuin-A by LC-MS/MS and immunoblot analysis (Fig. 1d). Fetuin-A was barely detectable in the eluate from the control beads. We also confirmed that alendronate bound to crystalline calcium-phosphate but not to amorphous calcium-phosphate (Fig. 1e). The result was the same when other bisphosphonates, such as neridronate, were used (data not shown), indicating that bisphosphonates can bind to calcium-phosphate crystals complexed with fetuin-A in plasma. Based on these observations, we conclude that the gel filtration method can quantify CPP containing crystalline calcium-phosphate.

To compare the sensitivity between the gel filtration method and the fetuin-A method, we measured CPP in archive serum samples from 68 dialysis patients using both methods. In the fetuin-A method, 24 out of the 68 patients (35%) exhibited the fetuin-A reduction ratio (RR) less than 2.2%. Because the %CV of the fetuin-A ELISA kit was 2.2%, serum CPP levels in these patients were considered below the detection limit. In contrast, the gel filtration method succeeded in measuring CPP levels in all the samples at the range of 10~400 fold higher than the background. Thus, we conclude that the gel filtration method is more sensitive than the fetuin-A method.

### Identification of a new class of CPP

**Figure 2:**
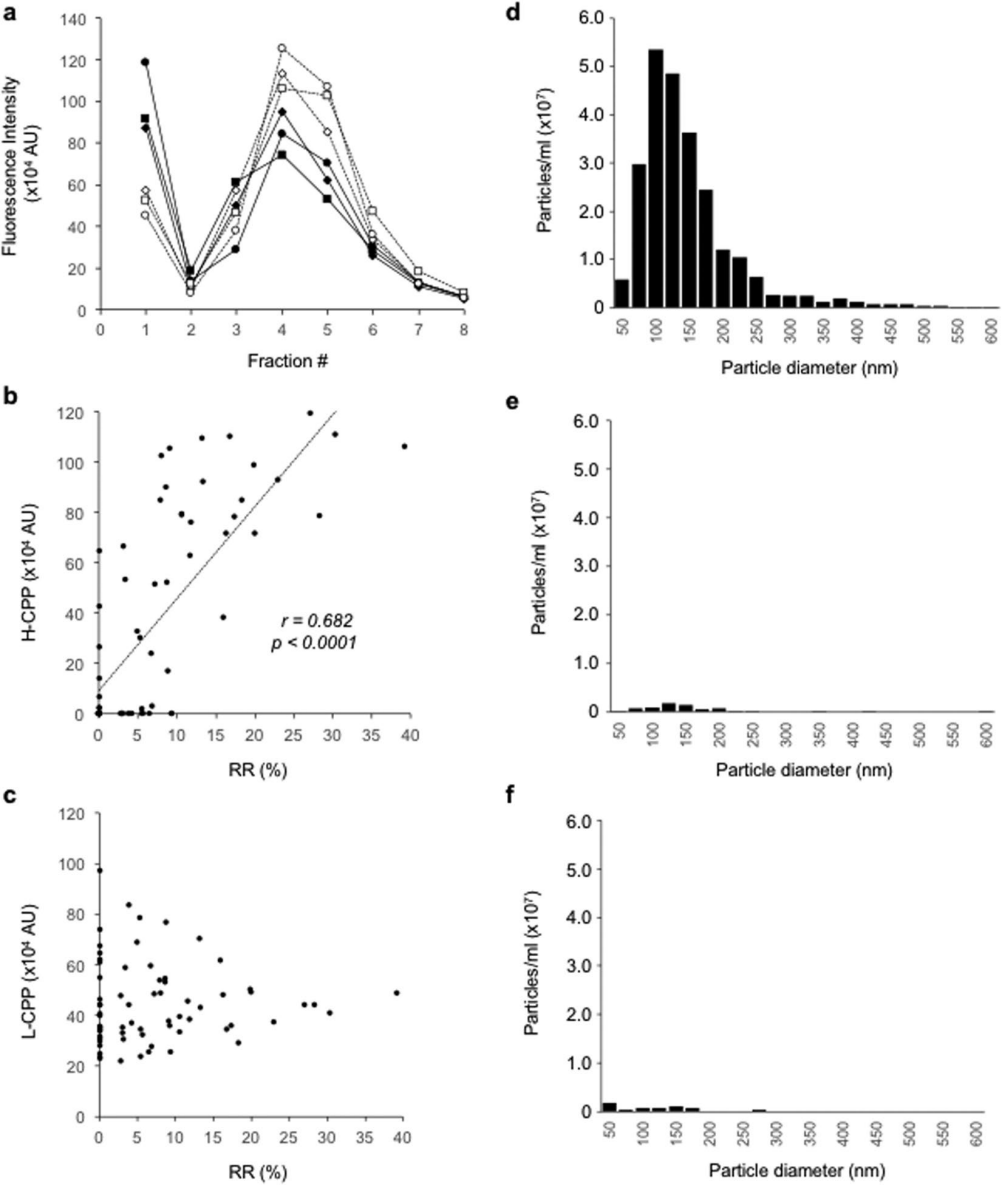
Identification and characterization of low-density CPP (L-CPP). (**a**) Gel filtration chromatograms of archive serum samples from three dialysis patients, which were centrifuged at 16,000 g for 2 hours (open markers with dot lines) or left untreated (closed markers with solid lines) before OsteoSense inoculation. (**b**) Comparison between the fetuin-A method and the gel filtration method. The CPP levels determined by the fetuin-A method were correlated with the H-CPP levels (**b**), but not with the L-CPP levels (**c**), determined by the gel filtration method. RR (%), the reduction rate (%) of serum fetuin-A levels. (**d**) The particle size distribution of CPP in one of the archive serum samples from dialysis patients determined by nanoparticle tracking analysis (NTA) using FITC-alendronate as a probe. (**e**) as in (**d**), except that EDTA was added to the serum sample before NTA. (**f**) as in (**d**), except that the serum sample was centrifuged at 16,000 g for 2 hours before NTA.

We measured CPP in the supernatant of the archive serum samples centrifuged at 16,000 g for 2 hours, which was supposed to contain few CPP, by the gel filtration method. However, unlike the EDTA treatment, the centrifugation failed to abolish the HMW peak, indicating that a decent amount of CPP remained in the supernatant (Fig. 2a). We designated those CPP that were not precipitated by centrifugation at 16,000 g for 2 hours as low-density CPP (L-CPP). The difference in the fluorescence intensity of the void fraction between before and after the centrifugation represented high-density CPP (H-CPP), which were precipitated by the centrifugation and thus considered equivalent to CPP measured by the fetuin-A method. In fact, serum CPP levels determined by the fetuin-A method were correlated with H-CPP levels (Fig. 2b), but not with L-CPP levels (Fig. 2c). To determine the particle size distribution of CPP, we added FITC-conjugated alendronate to the serum samples and then analyzed FITC-positive particles by nanoparticle tracking analysis (NTA). The diameter of CPP distributed predominantly between 50 and 250 nm (Fig. 2d). Almost all these CPP disappeared after the samples were treated with EDTA (Fig. 2e) or centrifuged at 16,000 g for 2 hours (Fig. 2f), indicating that the CPP detected in NTA are primarily H-CPP and that L-CPP are smaller than 50 nm in diameter and too small to be detected by NTA.

### Factors affecting quality and quantity of CPP *in vitro*

Before applying the gel filtration method to clinical settings, we needed to optimize and standardize a procedure of the assay, because various physicochemical factors potentially affect formation of calcium-phosphate crystals and phase transition from amorphous to crystalline calcium-phosphate *in vitro*, which can impact on the result. We evaluated effects of the following factors on total CPP, L-CPP, and H-CPP levels in subjects with normal renal function (control subjects) and in dialysis patients. 1) number of freeze-thaw cycles, 2) temperature at which samples are frozen/stored, 3) temperature at which samples are left before starting the assay, 4) time period for which samples are left before starting the assay, 5) composition of the buffer used for sample dilution, and 6) presence or absence of anti-coagulants. As shown below, two points have become clear. First, dialysis patients have higher total CPP, L-CPP, and H-CPP levels than control subjects in general. Second, samples from dialysis patients are affected more robustly by these factors than samples from control subjects.

**Figure 3:**
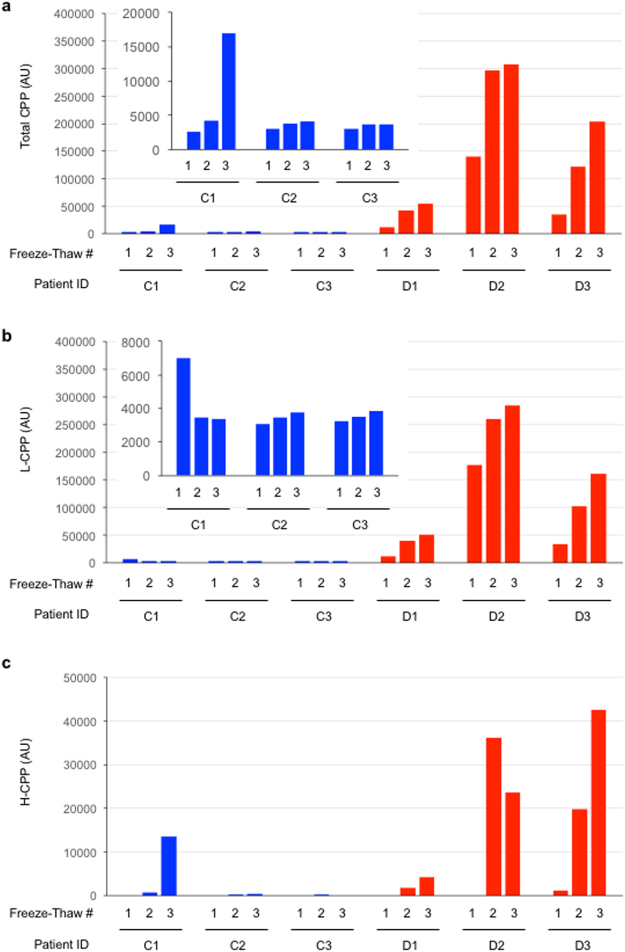
Effects of the number of sample freeze-thaw cycles on plasma CPP levels in three control subjects (C1, C2, and C3, indicated as blue bars) and in three dialysis patients (D1, D2, and D3, indicated as red bars). The insets are enlarged views of the control subjects. After blood sampling with heparin collecting tubes, plasma samples were separated within 60 minutes and aliquoted before snap-frozen in liquid nitrogen and stored at −80 °C. These samples were thawed at room temperature (one cycle of freeze-thaw) or snap-frozen in liquid nitrogen and then thawed again (two cycles of freeze-thaw). This process was repeated one more time (three cycles of freeze-thaw). The total CPP (**a**), L-CPP (**b**), and H-CPP (**c**) levels were shown.

As we increased the number of freeze-thaw cycles, total CPP and L-CPP levels were increased progressively in the dialysis patients (D1, D2, D3) and, albeit a smaller extent, in one of the three control subjects (C1). In contrast, repeated freeze-thaw had little effects in the other two control subjects (C2, C3) (Fig. 3a,b). Notably, H-CPP became detectable only after multiple cycles of freeze-thaw (Fig. 3c), indicating that H-CPP can be artifacts generated *in vitro* during sample processing. In fact, the increase in the total CPP in C1 was largely attributable to formation of H-CPP after three times of freeze-thaw.

**Figure 4:**
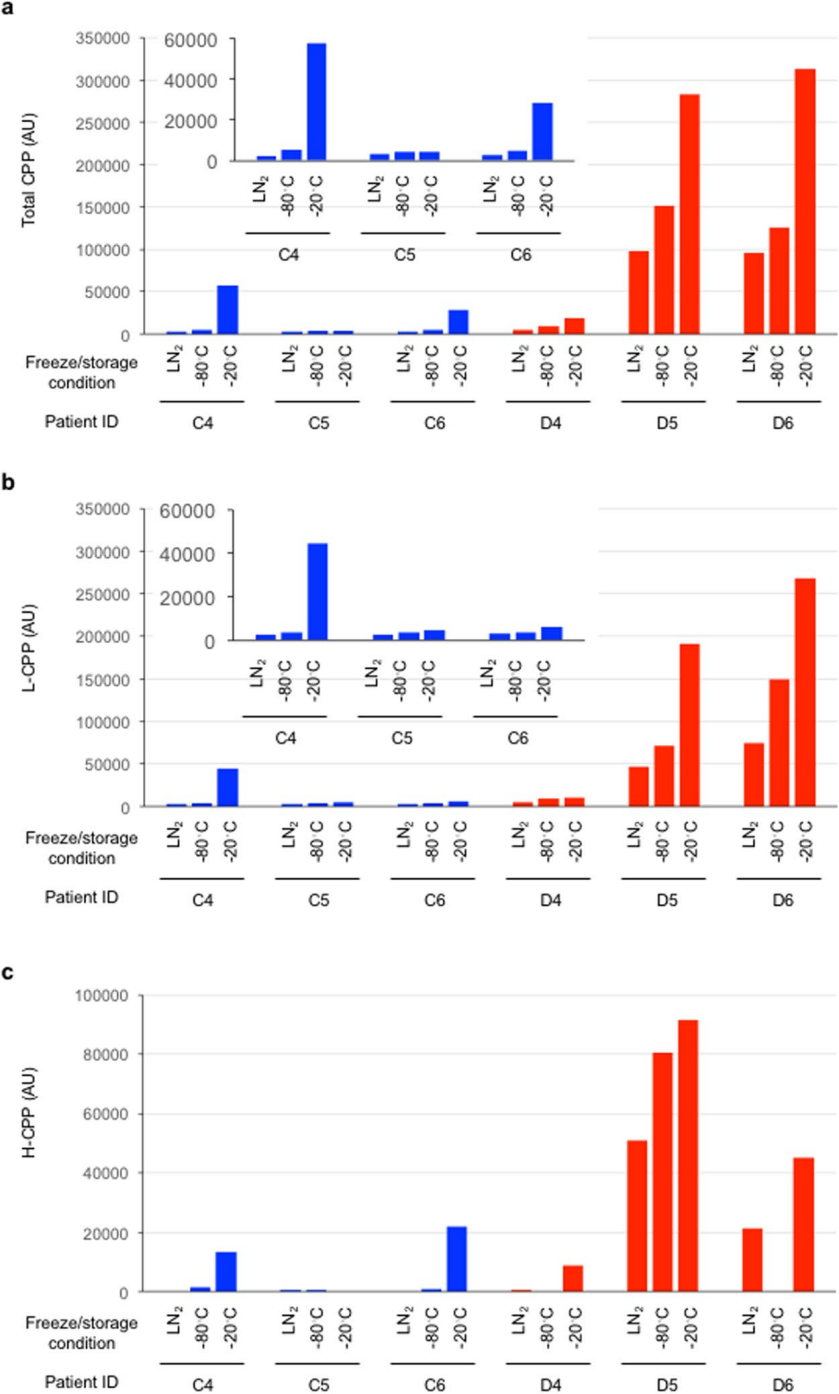
Effects of the speed of sample freezing on plasma CPP levels determined by the gel filtration method in three control subjects (C4, C5, and C6, indicated as blue bars) and in three dialysis patients (D4, D5, and D6, indicated as red bars). Plasma samples were snap-frozen in liquid nitrogen and then stored at −80 °C (LN_2_), or frozen at −80 °C and the stored at the same temperature (−80 °C), or frozen at −20 °C and then stored at the same temperature (−20 °C). The insets are enlarged views of the control subjects. The total CPP (**a**), L-CPP (**b**), and H-CPP (**c**) levels were shown.

Compared with plasma samples quickly frozen in liquid nitrogen and then stored at −80 °C, plasma samples frozen gradually and stored at −80 °C showed higher total CPP and L-CPP levels (Fig. 4a,b). These observations indicate that not only the storage temperature but also the speed of freezing influences CPP levels. Even higher total CPP and L-CPP levels were observed in plasma samples frozen slowly and stored at −20 °C (Fig. 4a,b). H-CPP levels showed the similar trend (Fig. 4c).

**Figure 5:**
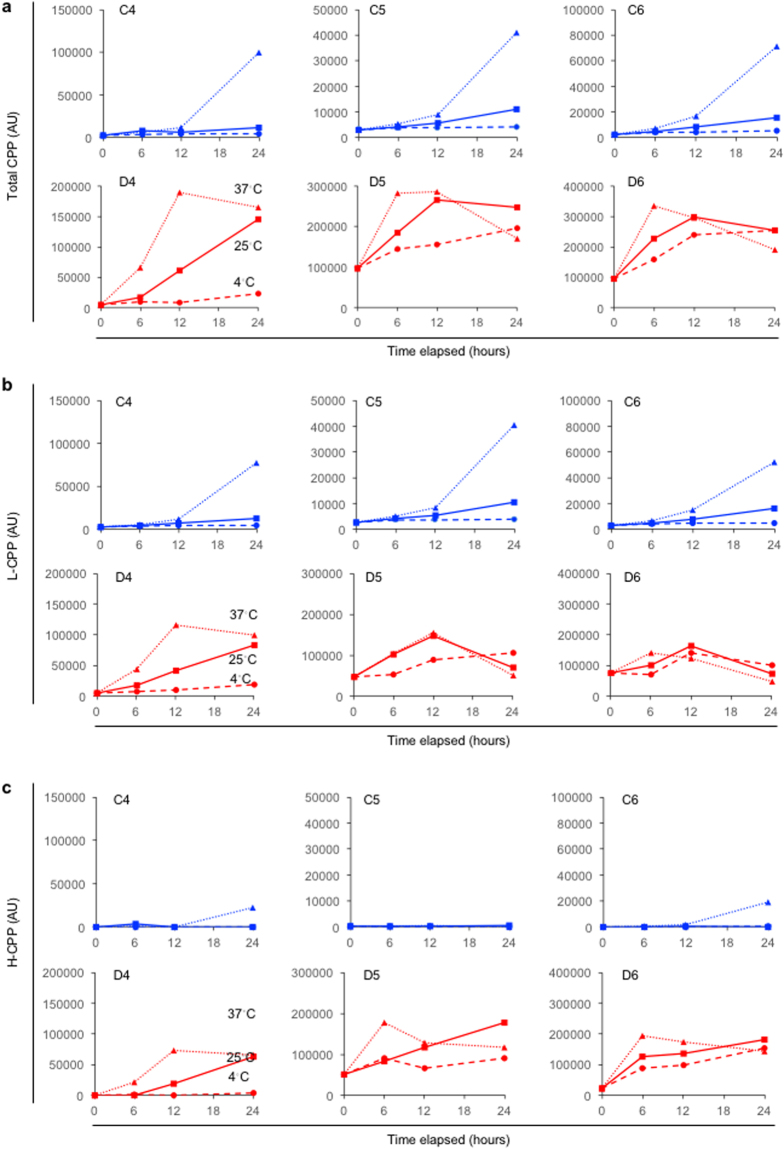
Effects of the time duration and temperature after thawing the plasma samples on CPP levels in three control subjects (C4, C5, and C6, indicated as blue lines) and three dialysis patients (D4, D5, and D6, indicated as red lines). All the plasma samples were snap-frozen in liquid nitrogen and stored at −80 °C until thawed for the CPP assay. The thawed samples were incubated at 4 °C, 25 °C, or 37 °C for 0, 6, 12, or 24 hours before measuring total CPP (**a**), L-CPP (**b**), and H-CPP (**c**) levels.

It is often the case in clinical settings that samples are left for hours on a bench or in a refrigerator until subjected to the assay. We found that total CPP, L-CPP, and H-CPP levels were increased in a time- and temperature-dependent manner in the control subjects (Fig. 5, blue). The time course of the change in CPP levels were accelerated in dialysis patients, as the increase became evident within 12 hours at 25 °C and within 6 hours at 37 °C in general (Fig. 5, red). However, their CPP levels were not increased or rather decreased after that for unknown reasons. At baseline (at the time point of zero), the total CPP and L-CPP levels in the dialysis patients were already higher than those in the control subjects. This may be partly because the CPP levels in the dialysis patients were increased more robustly than those in the control subjects during the assay procedure, which includes incubation at 25 °C for 60 minutes with OsteoSense. Another reason may lie in the fact that all the samples used in Fig. 5 were not fresh but stored plasma samples that had undergone one-cycle of freeze-thaw. This may also be a reason that H-CPP were detectable in two of the three dialysis patients (D5 and D6) at baseline.

**Figure 6:**
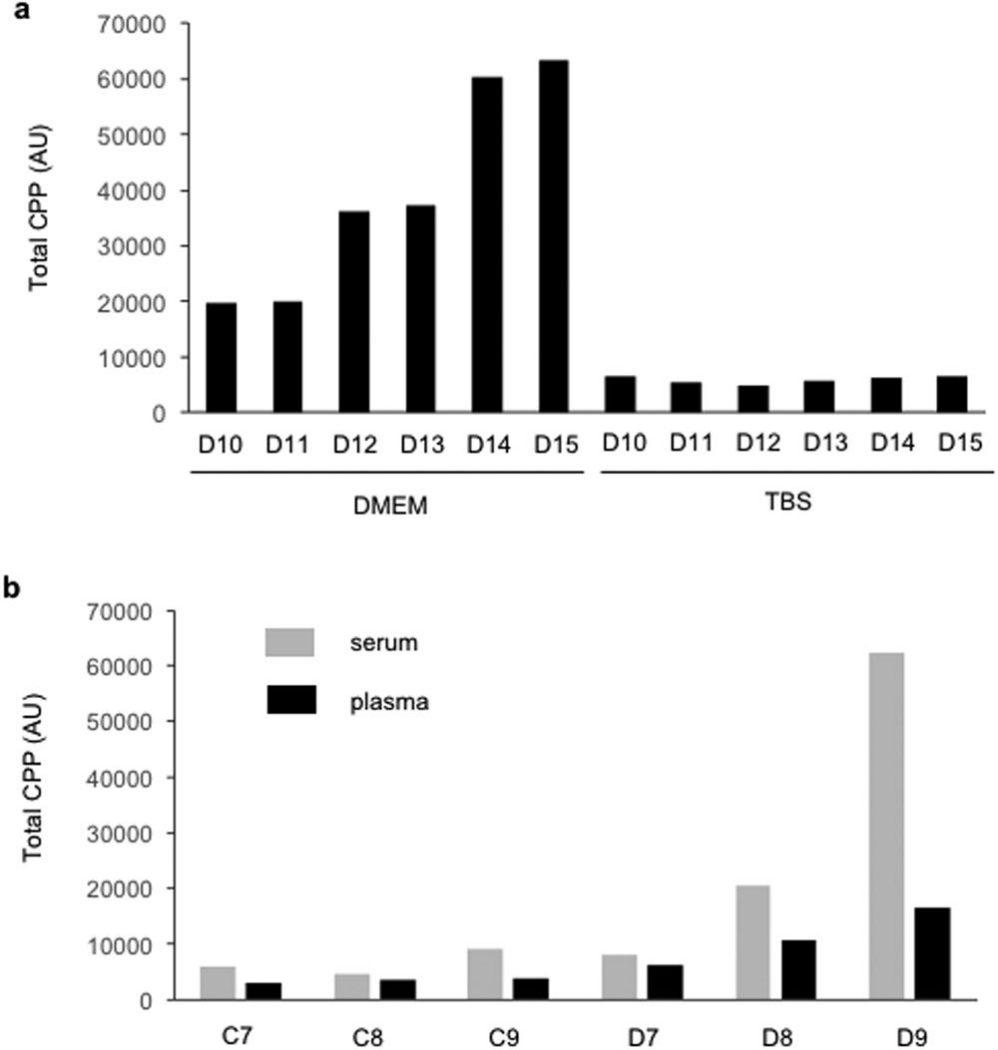
Effects of additives to plasma samples on CPP levels. (**a**) Plasma samples from six dialysis patients (D10-D15) were diluted 10 fold with tissue culture medium (DMEM) or Tris-buffered saline (TBS) for measuring total CPP levels by the gel filtration method. The gel filtration method normally uses DMEM, but not TBS, as the diluent. (**b**) Both serum and heparin plasma were prepared from the same blood samples in three control subjects (C7, C8, and C9) and three dialysis patients (D7, D8, and D9). The serum and plasma samples were snap-frozen in liquid nitrogen and stored at −80 °C until total CPP levels were measured by the gel filtration method.

Composition of the buffer used for diluting samples also affect CPP levels. Total CPP levels were decreased when plasma samples were suspended in the buffer low in the calcium and phosphate concentration like Tris-buffered saline (TBS) (Fig. 6a**)**, likely because calcium-phosphate crystals were dissolved. We also found that the serum CPP level was consistently higher than the plasma CPP level (Fig. 6b). Based on these observations, we conclude that CPP in stored plasma or serum samples are different from the CPP circulating in the blood.

### CPP in fresh plasma

**Figure 7:**
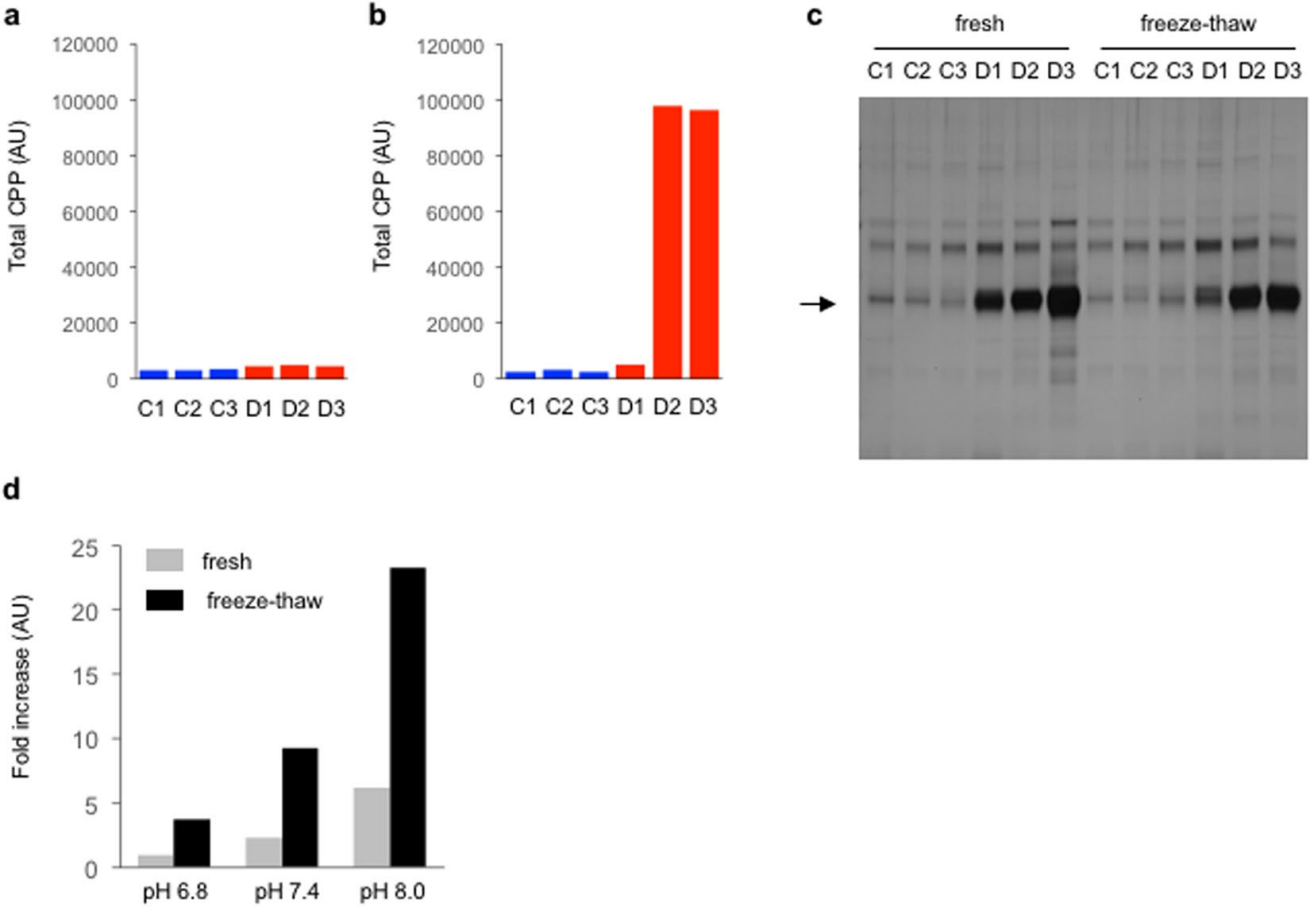
CPP in fresh plasma. Total CPP levels were measured in plasma samples from three control subjects (C1, C2, and C3, indicated as blue bars) and three dialysis patients (D1, D2, and D3, indicated as red bars). (**a**) Total CPP in fresh plasma. (**b**) Total CPP in the plasma samples frozen and thawed once. The aliquots of the fresh plasma samples used in (**a**) were snap-frozen in liquid nitrogen and stored at −80 °C until thawed for the CPP assay. (**c**) Pull-down of CPP from the fresh plasma and the freeze-thawed plasma samples using alendronate beads as in Fig. 1c. The arrow indicates fetuin-A. The full-length gel was shown in Supplementary information. (**d**) The transition of amorphous calcium-phosphate to crystalline calcium-phosphate induced by freeze-thaw. Nanoparticles of amorphous calcium-phosphate were suspended in DMEM with different pH (6.8, 7.4, and 8.0). The binding capacity of OsteoSense to the particles were quantified before and after one cycle of freeze-thaw.

To avoid possible (trans)formation of CPP during sample preparation/storage *in vitro* and gain the best estimation of CPP *in vivo*, we measured CPP levels in fresh plasma. Immediately after blood sampling, heparin plasma was prepared from three dialysis patients and three control subjects and promptly measured CPP by the gel filtration method. Total CPP levels in these fresh plasma samples were not very different between dialysis patients and control subjects (Fig. 7a). H-CPP were undetectable (data not shown). These findings indicate that L-CPP were the primary form of CPP circulating in the blood and that the amount of crystalline calcium-phosphate was comparable between the dialysis patients and the control subjects. However, after the samples were snap-frozen in liquid nitrogen and thawed once, total CPP levels were increased significantly in two out of the three dialysis patients but not in the control subjects (Fig. 7b).

To explain these findings, we considered two possible mechanisms, which were not mutually exclusive. First, in plasma samples from the two dialysis patients (D2, D3), freeze-thaw promoted formation of new CPP *in vitro*. Second, freeze-thaw induced transition of calcium-phosphate from the amorphous phase to the crystalline phase to increase OsteoSense binding. To determine which (or both) was the case, we first tested whether freeze-thaw would increase the amount of fetuin-A pulled down with alendronate beads. As shown in Fig. 7c, freeze-thaw never promoted formation of new CPP *in vitro* in all the samples. We next tested whether freeze-thaw would induce the amorphous to crystalline phase transition of calcium-phosphate. We dispersed amorphous calcium-phosphate nanoparticles in Krebs-Ringer-HEPES (KRH) with three different pH and determined the binding capacity of OsteoSense to the nanoparticles before and after freeze-thaw. Several fold increase in OsteoSense binding was observed after one cycle of freeze-thaw, indicating that freeze-thaw promoted the phase transition of amorphous calcium-phosphate into crystalline calcium-phosphate *in vitro* (Fig. 7d).

**Figure 8:**
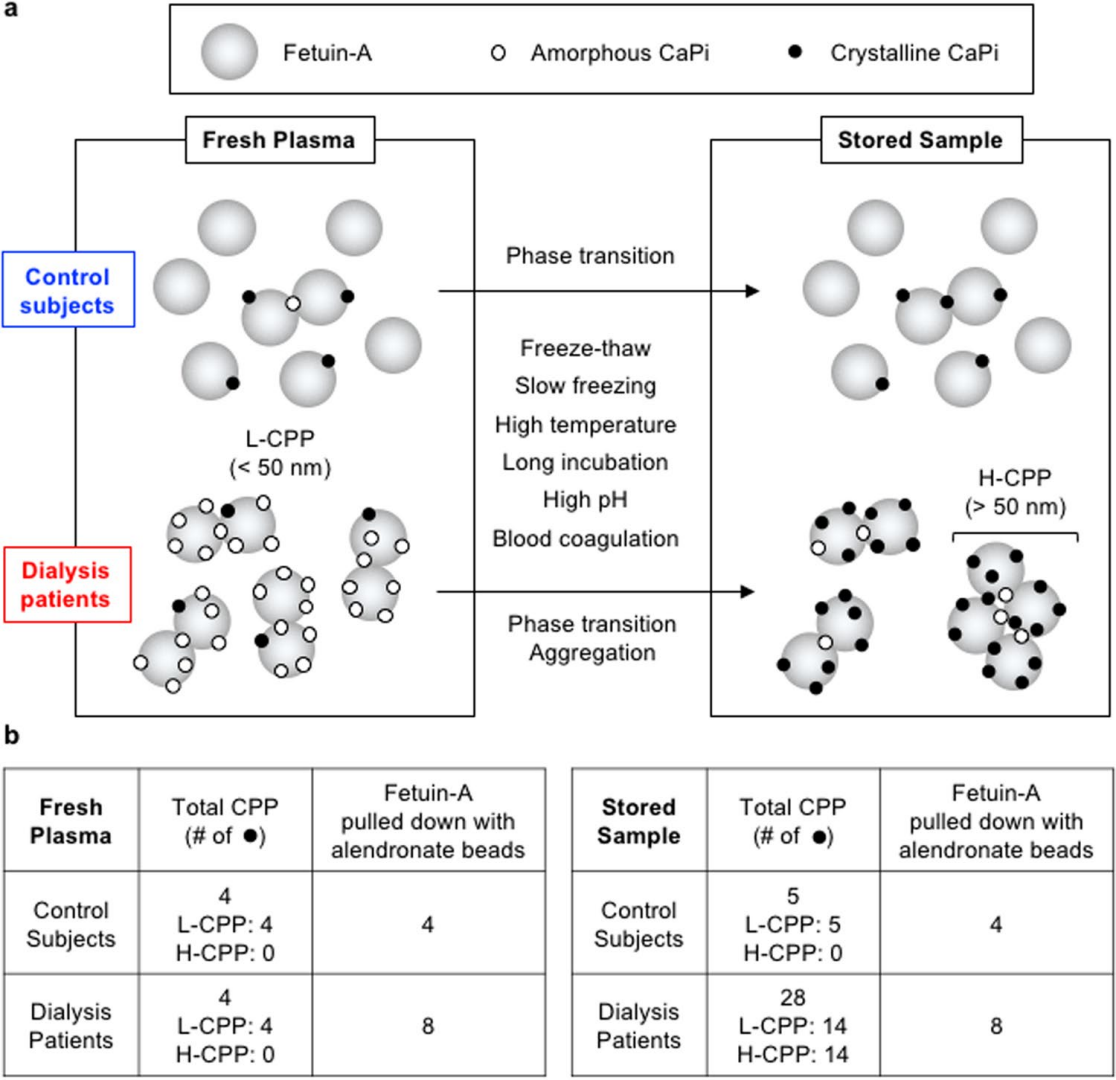
A proposed model of CPP and transformation *in vitro*. (**a**) A schematic representation of CPP in fresh and stored samples. For simplicity, L-CPP and H-CPP were depicted as monomers/dimers and tetramers of fetuin-A molecules (large grey circles), respectively, laden with tiny calcium-phosphate (CaPi) particles, which are either amorphous CaPi (open circles) or crystalline CaPi (closed circles) or both. In response to indicated physicochemical conditions during sample processing *in vitro*, CaPi in fresh plasma undergo the amorphous to crystalline phase transition. In addition, L-CPP aggregate to form H-CPP, which occurs preferentially in dialysis patients. (**b**) Predicted relative levels of CPP determined by the gel filtration method and fetuin-A pulled down with alendronate beads in the model, which coincide with the actual data.

It should be noted that alendronate beads consistently pulled down more fetuin-A from fresh plasma of the dialysis patients than from that of the control subjects (Fig. 7c), despite the fact that the total CPP levels determined by the OsteoSense binding capacity were not very different (Fig. 7a). We repeated these experiments using more than 10 fresh plasma samples from dialysis patients and control subjects to observe the same results (data not shown). This discrepancy has prompted us to speculate the difference in the CPP composition between dialysis patients and control subjects (Fig. 8a). The dialysis patients had higher numbers of L-CPP, each carrying higher numbers of calcium-phosphate particles than the control subjects. However, L-CPP in the dialysis patients were rich in amorphous calcium-phosphate and thus underestimated by the gel filtration method, which can measure only CPP carrying crystalline calcium-phosphate. In addition, each of the L-CPP in the dialysis patients contained higher numbers of fetuin-A molecules than that in the control subjects. This model explains why CPP levels in fresh plasma determined by the gel filtration method were not very different between the dialysis patients and the control subjects (Fig. 7a), why the amount of fetuin-A pulled down with alendronate beads was more abundant in the dialysis patients than in the control subjects (Fig. 7c), and why CPP levels were increased more robustly in dialysis patients than in control subjects by freeze-thaw and other physicochemical stimuli (Figs 3, 4, 5, 7b and 7d) that potentially promote transition of calcium-phosphate from the amorphous phase to the crystalline phase (Fig. 8b)^10^.

### Association between CPP levels and clinical parameters in CKD patients

These observations indicate that CPP levels in fresh plasma determined by the gel filtration method do not necessarily reflect the amounts of CPP in the sample. In addition, it is practically difficult to measure CPP levels in fresh plasma in a large number of patients. Therefore, we have standardized a condition for sample preparation that is reasonably attainable in clinical settings and still capable of estimating the total amounts of CPP by adequately inducing the amorphous to crystalline transition. Blood samples collected with heparin blood collecting tubes were centrifuged to separate plasma within 60 minutes after sampling. The plasma samples were aliquoted in microcentrifuge tubes, snap-frozen in liquid nitrogen, and stored at −80 °C. The frozen plasma samples were thawed 24 hours before starting the CPP assay and incubated at 25 °C to induce the phase transition of the amorphous calcium-phosphate in CPP to crystalline calcium-phosphate to make them detectable by OsteoSense. After 22 hours, each sample was divided into two tubes. One of the tubes was centrifuged at 16,000 g for 2 hours at 25 °C to be used for measurement of the L-CPP level. The other tube was left untreated at 25 °C for 2 hours and used for measurement of the total CPP level. The H-CPP level was calculated by subtracting the L-CPP level from the total CPP level. We also measured several blood parameters possibly associated with serum CPP levels, including phosphate, calcium, magnesium, estimated glomerular filtration rate (eGFR), intact parathyroid hormone (iPTH), fibroblast growth factor-23 (FGF23), and 1,25-dihydroxyvitamin D_3_ (1,25(OH)_2_D_3_).

We analyzed plasma samples from 148 pre-dialysis CKD patients recruited at Jichi Medical University Hospital, which were composed of 11 stage 1, 30 stage 2, 63 stage 3, 29 stage 4, and 15 stage 5 patients (Table 1). Univariate analysis revealed that total CPP levels were associated positively with age, FGF23, and phosphate, but negatively with eGFR and 1,25(OH)_2_D_3_ (Table 2). No significant association was observed with serum calcium, magnesium, and iPTH levels. To identify factors that account total CPP levels, we performed multiple regression analysis by entering those factors significantly associated in the univariate analysis into the final multivariable model simultaneously and identified age and serum phosphate as independent explanatory variables of the total CPP (adjusted R^2^ = 0.604, *P* < 2.2 × 10^−16^, Table 2). Serum phosphate was the major determinant of total CPP (standardized partial regression coefficient = 0.805). L-CPP levels were strongly correlated with total CPP levels (r = 0.981, 95%CI: 0.974–0.986, p < 2 × 10^−16^) and, like total CPP, positively associated with age, FGF23, and phosphate, but negatively with eGFR and 1,25(OH)_2_D_3_. In addition to age and phosphate, multiple regression analysis identified FGF23 as independent explanatory variables of L-CPP levels (adjusted R^2^ = 0.583, *P* < 2.2 × 10^−16^, Table 3). Again, Serum phosphate was the major determinant of L-CPP (standardized partial regression coefficient = 0.808).

**Table 1:** The profile of serum and plasma parameters measured in the 148 non-dialysis patients (44 females and 104 males). Ca, calcium; eGFR, estimated glomerular filtration rate; FGF23, fibroblast growth factor-23; iPTH, intact parathyroid hormone; Mg, magnesium; 1,25(OH)_2_D_3_, 1,25-dihydroxyvitamin D_3_; P, phosphate.

Variable (Unit)	Mean (25–75%)
age (years)	63.0 (56–74)
serum Ca (mg/dl)	9.35 (9.1–9.7)
eGFR (ml/min/1.73m^3^)	45.9 (27.8–63.5)
serum FGF23 (pg/ml)	99.1 (44.1–92.9)
serum iPTH (pg/ml)	81.6 (36–80)
serum Mg (mg/dl)	2.16 (1.9–2.2)
serum 1,25(OH)_2_D_3_ (pg/ml)	42.3 (28.3–52.0)
serum P (mg/dl)	3.37 (2.9–3.6)
plasma total CPP (AU)	60640 (11209–81760)
plasma L-CPP (AU)	55330 (11771–86949)

**Table 2:** Association of total CPP levels with clinical parameters in 148 non-dialysis CKD patients. See Table 1 for abbreviations. r, Pearson’s correlations; β, standardized partial regression coefficient. The adjusted R^2^ is 0.604.

Variable (Unit)	Univariate		Multivariate	
	r	p	β	p
Age (years)	0.274	0.0007	0.155	0.011
serum Ca (mg/dl)	0.108	0.192	—	—
eGFR (ml/min/1.73m^3^)	−0.311	0.0001	0.060	0.480
serum FGF23 (pg/ml)	0.316	9.36 × 10^−5^	−0.162	0.054
serum iPTH (pg/ml)	0.139	0.096	—	—
serum Mg (mg/dl)	0.129	0.119	—	—
serum 1,25(OH)_2_D_3_ (pg/ml)	−0.256	0.0017	−0.110	0.109
serum P(mg/dl)	0.796	1.4 × 10^−33^	0.805	<2 × 10^−16^

**Table 3:** Association of L-CPP levels with clinical parameters in 148 non-dialysis CKD patients. See Table 2 for abbreviations. The adjusted R^2^ is 0.583.

Variable (Unit)	Univariate		Multivariate	
	r	p	β	p
Age (years)	0.277	0.0006	0.142	0.024
serum Ca (mg/dl)	0.155	0.0598	—	—
eGFR (ml/min/1.73 m^3^)	−0.296	0.0003	0.031	0.720
serum FGF23 (pg/ml)	0.300	0.0002	−0.206	0.017
serum iPTH (pg/ml)	0.098	0.242	—	—
serum Mg (mg/dl)	0.139	0.092	—	—
serum 1,25(OH)_2_D_3_ (pg/ml)	−0.231	0.0047	−0.103	0.144
serm P (mg/dl)	0.798	6.4 × 10^−34^	0.808	<2 × 10^−16^

H-CPP levels were measurable only in 49 out of the 148 patients. These 49 patients who had measurable levels of H-CPP (H-CPP holders) had significantly lower eGFR, higher FGF23, higher iPTH, lower 1,25(OH)_2_D_3_, higher phosphate, and higher total CPP and L-CPP levels than the 99 patients who had undetectable levels of H-CPP (H-CPP non-holders) (Table 4). Because strong colinearity was observed between total CPP and L-CPP levels, we modeled total CPP and L-CPP separately in multivariable-adjusted logistic regression analysis. Among these factors, only total CPP remained as the independent explanatory variable of the H-CPP holders (odds ratio = 9.51, 95%CI: 2.48–36.5, p = 0.001). In fact, H-CPP levels were strongly correlated with total CPP levels (r = 0.957, 95% CI: 0.926–0.975, p < 2 × 10^−16^) among the H-CPP holders.

**Table 4:** Comparison of clinical parameters between H-CPP holders and non-holders. Statistical difference was evaluated by Mann-Whitney U-test. See Table 1 for abbreviations.

Variable (Unit)	Median (25–75%)		p
	H-CPP = 0 (*N* = 99)	H-CPP > 0 (*N* = 49)	
Age (years)	62.0 (55.5–73.0)	68.0 (58.0–76.0)	0.0669
serum Ca (mg/dl)	9.4 (9.1–9.7)	9.3 (9.0–9.6)	0.2450
eGFR (ml/min/1.73m^3^)	46.0 (30.0–69.5)	33.0 (20.0–52.0)	0.0158
serum FGF23 (pg/ml)	59.9 (43.6–80.5)	82.4 (49.2–122.0)	0.0095
serum iPTH (pg/ml)	42.5 (31.6–69.5)	60.0 (43.0–109.0)	0.0038
serum Mg (mg/dl)	2.1 (1.9–2.2)	2.1 (1.9–2.4)	0.3910
serum 1,25(OH)_2_D_3_ (pg/ml)	42.0 (31.6–55.5)	38.0 (24.8–48.5)	0.0194
serum P (mg/dl)	3.2 (2.9–3.4)	3.5 (3.1–4.1)	0.0005
Total CPP (AU)	17404 (10127–47393)	66053 (27852–163121)	1.87 × 10^–6^
L-CPP (AU)	19692 (11234–60716)	55781 (25553–115205)	0.0015

## Discussion

Using the gel filtration method, we have identified L-CPP, which are smaller in size and lower in density than CPP described in previous studies. The CPP detected by the fetuin-A method and the flow cytometric method are equivalent to H-CPP in the gel filtration method in that they are precipitated by centrifugation at 16,000 ~ 24,000 g for 2 hours^4,8,9^. It should be noted that L-CPP and H-CPP are distinct from primary CPP (a.k.a. CPP-I) and secondary CPP (a.k.a. CPP-II)^4,6,11^, respectively. Primary CPP and secondary CPP are generated *in vitro* by adding calcium and phosphate to diluted serum to increase their concentrations to supra-physiological levels^4^. Alternatively, CPP-I and CPP-II are isolated from uremic patients by centrifugation at 22,000 g for 2 hours and washed with TBS followed by affinity purification with a fetuin-A antibody^6,11^. In either case, these CPP have experienced significant changes in calcium and phosphate concentrations in the dispersion medium, and therefore, potentially differ from their native forms *in vivo* or reflect a particular fraction of CPP that survived these changes in calcium and phosphate levels. In contrast, L-CPP and H-CPP are present and/or generated in straight plasma without drastic changes in the calcium and phosphate levels, and thus likely different from primary CPP and secondary CPP regarding the physical property and chemical composition. In fact, both primary CPP and secondary CPP have the density comparable with H-CPP, because both can be precipitated by centrifugation at 16,000~24,000 g for 2 hours. The particle size of primary CPP and secondary CPP are reportedly between 80 nm and 250 nm in diameter^4,11^, which is also comparable with H-CPP (Fig. 2d). Hence, primary CPP and secondary CPP are similar to H-CPP in terms of their physical property. Regarding their chemical composition, however, calcium-phosphate in primary CPP is thought amorphous but not crystalline^4^. Because bisphosphonates barely bind to amorphous calcium-phosphate (Fig. 1e), primary CPP are not supposed to bind to OsteoSense or pulled down with the alendronate beads, and thus different from L-CPP and H-CPP.

Theoretically, it is possible for the gel filtration method to detect two kinds of particles in the blood other than CPP. One is exosomes or microvesicles carrying calcium-phosphate crystals. Although these crystals are surrounded by the plasma membrane, they may be labeled with OsteoSense, if it penetrates the membrane or if the membrane has an open seam. However, in the pull-down experiments of plasma using alendronate beads (Fig. 1c,d), immunoblot analysis failed to detect annexin V in the eluate (data not shown), a marker of exosomes or microvesicles, indicating that contribution of such membrane-associated calcium-phosphate crystals to the CPP level determined by the gel filtration method may be minimal. The other is inorganic crystalline calcium-phosphate particles with a molecular weight higher than the molecular weight cut-off of the gel filtration spin column (40 kDa in this study). Such inorganic calcium-phosphate particles, if any, should contribute to L-CPP levels. Because they are protein free, it may be nominally inadequate to call them as calciprotein particles. However, it is considered clinically meaningful to take such inorganic calcium-phosphate nanocrystals into account, because they are more potent than CPP in the ability to induce inflammatory and apoptotic responses in cultured macrophages^6^, and thus predicted as more pathogenic than CPP. Further investigations are required to prove or disprove the presence of protein-free, calcium-phosphate nanocrystals in the blood.

We have shown in the present study that CPP in stored serum/plasma samples can be transformed *in vitro* after blood sampling. Assuming that CPP in fresh plasma are the best estimation of CPP *in vivo*, CPP circulating in the blood are primarily L-CPP. However, various physicochemical stimuli during sample preparation can induce aggregation of L-CPP to generate H-CPP and transition of calcium-phosphate from the amorphous phase to the crystalline phase to increase OsteoSense binding (Fig. 8). Therefore, characteristics of CPP in stored serum samples described in previous studies are likely dissimilar to those *in vivo*. For example, observation of purified CPP from patients’ serum under electron microscopy (EM) requires CPP to be precipitated by centrifugation for 2 hours, purified by affinity chromatography, re-suspended in buffer, and placed in a vacuum or cryo-chamber for EM observation^6^. This sample processing entails multiple steps that potentially alter CPP both in quality and in quantity. Specifically, the time duration for the sample preparation, which took many hours, was sufficient to induce aggregation of L-CPP and formation of H-CPP (Fig. 5). If the CPP were suspended in a buffer containing calcium and phosphate, evaporation of water in vacuum must have increased their concentration and caused precipitation of calcium-phosphate on the EM stage. If the CPP were suspended in a buffer free from calcium and phosphate such as Tris-buffered saline (TBS)^6^, CPP must have been dissolved (Fig. 6b). Because the flow cytometric method used TBS to suspend CPP before sorting^9^, it is likely that the majority of CPP had been dissolved and that a particular fraction of mineral-containing nanoparticles insoluble with TBS was analyzed. Thus, in order to resolve the physical property, chemical composition, and structure of CPP representing those actually circulating in the blood, it is necessary to develop a new analytical method in which any possible risk for altering CPP *in vitro* is carefully eliminated. Also, in basic research to investigate the activity of CPP using cultured cells, one should be aware that CPP in the medium are subject to change depending on various experimental conditions, including incubation time, temperature, pH, and composition of the medium, which potentially influence the physical property and thus activity of CPP.

**Figure 9:**
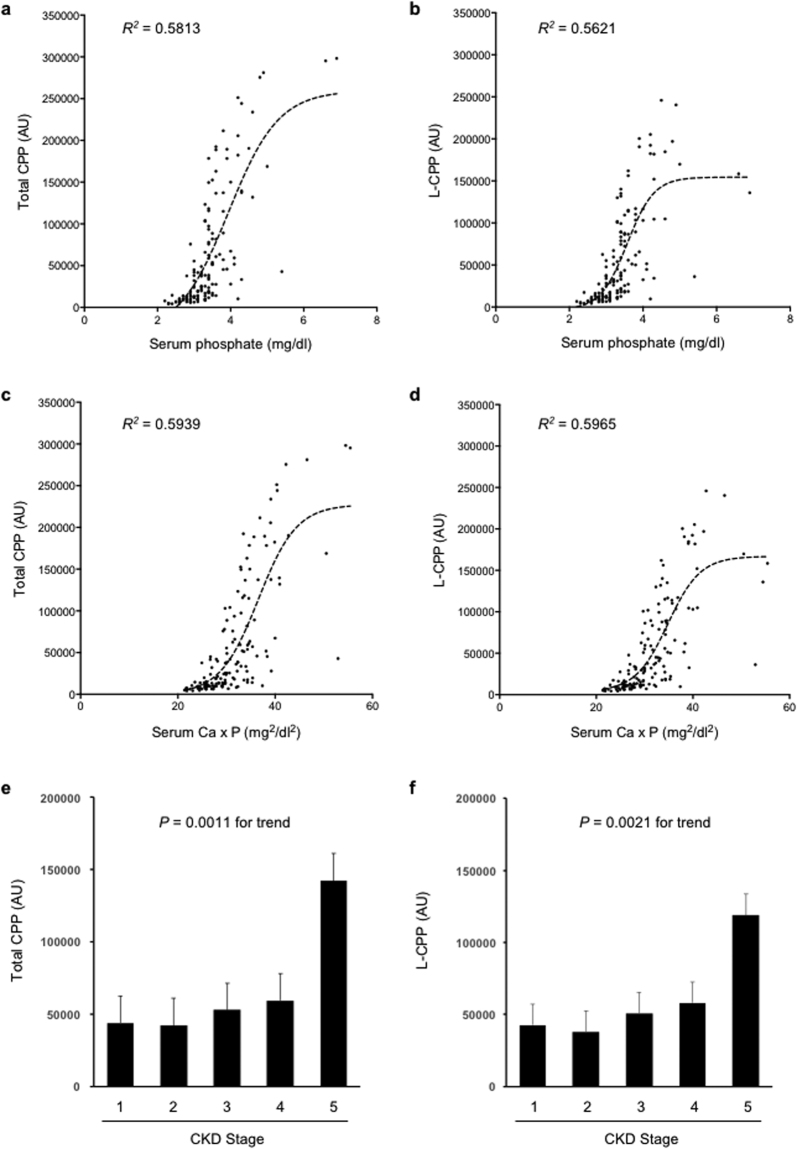
Plasma total CPP and L-CPP levels in 148 non-dialysis patients. Correlation of serum phosphate levels with total CPP (**a**) or L-CPP (**b**) is shown with sigmoidal curve fitting (*R*^2^ = 0.5813 or 0.5621, respectively). Note that the normal range of serum phosphate levels are between 2.5 and 4.5 mg/dl. Correlation of serum calcium phosphate products with total CPP (**c**) or L-CPP (**d**) is shown with sigmoidal curve fitting (*R*^2^ = 0.5939 or 0.5965, respectively). Total CPP (**e**) and L-CPP (**f**) levels were increased with CKD progression. CKD stages were determined based on eGFR (ml/nin/1.73 m^2^), namely, stage 1: ≥90, stage 2: 60–89, stage 3: 59–30, stage 4: 15–29, stage 5: < 15.

It is of particular interest that total CPP and L-CPP levels in pre-dialysis CKD patients are independently associated with age and serum phosphate levels (Tables 2 and 3). Regarding the link between aging and phosphate, Tonelli and his colleagues analyzed 4,127 patients with hyperlipidemia and a history of myocardial infarction and identified a graded and independent association of serum phosphate levels with all-cause mortality^12^. Of note, the majority (94.2%) of the patients had normal serum phosphate levels (2.5–4.5 mg/dl). Although the mechanism behind this link remains to be determined, the fact that serum phosphate levels within normal range correlate independently with total CPP and L-CPP levels (Fig. 9a,b, Tables 2 and 3) may provide a clue. Patients with higher phosphate levels have higher CPP levels that potentially exacerbate arteriosclerosis and chronic non-infectious inflammation as discussed earlier, which may accelerate age-related pathologies to increase all-cause mortality. Namely, CPP may be regarded as a causative reagent that increases mortality with age. In support of this notion, multivariate analysis of the pre-dialysis CKD patients identified total CPP as a major independent explanatory variable of age besides eGFR. Standardized partial regression coefficient of eGFR and total CPP are −0.510 and 0.343, respectively (Table 5). Comparably, standardized partial regression coefficient of eGFR and L-CPP are −0.495 and 0.346, respectively (Table 6).

**Table 5:** Association of age with clinical parameters in 148 non-dialysis CKD patients. See Table 2 for abbreviations. Those factors that are significantly associated in the univariate analysis are entered simultaneously into the final multivariable model (The adjusted R^2^ = 0.298, *P* = 8.4 × 10^−10^). Because total CPP and L-CPP showed strong colinearlity, L-CPP was not included in the final multivariable model.

Variable (Unit)	Univariate		Multivariate	
	r	p	β	p
serum Ca (mg/dl)	−0.229	0.0052	−0.171	0.036
eGFR (ml/min/1.73 m^3^)	−0.446	1.32 × 10^−8^	−0.510	2.87 × 10^–6^
serum FGF23 (pg/ml)	0.168	0.0412	−0.264	0.021
serum iPTH (pg/ml)	0.206	0.0131	−0.009	0.935
serum Mg (mg/dl)	0.251	0.0021	0.167	0.023
serum 1,25(OH)_2_D_3_ (pg/ml)	−0.143	0.0836	—	—
serum P (mg/dl)	0.228	0.0053	−0.147	0.279
Total CPP (AU)	0.300	0.0002	0.343	0.004

**Table 6:** Association of age with clinical parameters in 148 non-dialysis CKD patients. See Table 2 for abbreviations. Those factors that are significantly associated in the univariate analysis are entered simultaneously into the final multivariable model (The adjusted R^2^ = 0.297, *P* = 8.9 × 10^–10^). Because total CPP and L-CPP showed strong colinearlity, total CPP was not included in the final multivariable model.

Variable (Unit)	Univariate		Multivariate	
	r	p	β	p
serum Ca (mg/dl)	−0.229	0.0052	−0.189	0.022
eGFR (ml/min/1.73m^3^)	−0.446	1.32 × 10^−8^	−0.495	6.67 × 10^−6^
serum FGF23 (pg/ml)	0.168	0.0412	−0.253	0.028
serum iPTH (pg/ml)	0.206	0.0131	0.002	0.986
serum Mg (mg/dl)	0.251	0.0021	0.171	0.021
serum 1,25(OH)_2_D_3_ (pg/ml)	−0.143	0.0836	—	—
serum P (mg/dl)	0.228	0.0053	−0.151	0.137
L-CPP (AU)	0.292	0.0003	0.346	0.005

Of note, the sigmoidal relation between the serum phosphate and plasma CPP levels indicates that a small increase in serum phosphate, even within the normal range, can cause a substantial increase in CPP (Fig. 9a,b). This finding may justify clinical studies to prove that phosphate-lowering therapies, either administration of phosphate binders or restriction of dietary phosphate intake or both, may reduce plasma CPP levels and improve some clinical outcomes of CKD patients without hyperphosphatemia. If proven, the new KDIGO guideline for the treatment of CKD may need to be revisited, which recommends phosphate-lowering therapies to be applied only when serum phosphate levels exceed the normal range^13^.

Also of note, the calcium phosphate products show a better fit with CPP levels than serum phosphate levels (Fig. 9c,d), which is reasonable because increase in either calcium or phosphate can facilitate precipitation of calcium-phosphate. Consistent with the previous reports^7^, plasma CPP levels increase with CKD progression (Fig. 9e,f).

In summary, we have developed a novel assay for measuring CPP levels and identified a new class of CPP (L-CPP). L-CPP are smaller in size and lower in density than the CPP described previously. Because CPP in fresh plasma are almost exclusively L-CPP, we reason that L-CPP are the major form of CPP *in vivo* and that the other forms of CPP found in stored samples are artifacts generated *in vitro* after blood sampling. Because various physicochemical factors during sample processing induce CPP transformation *in vitro*, such as aggregation of L-CPP to form H-CPP and transition of calcium-phosphate from the amorphous phase to the crystalline phase, it is imperative for any clinical studies on CPP to maintain consistency in the entire process from blood sampling through the CPP assay. Further investigations on the structure and biological activity of L-CPP are required to determine whether L-CPP may contribute to pathogenesis of age-related disorders.

## Methods

### CPP assay

Five μl of serum or plasma was added to 45 μl of Dulbecco’s Modified Eagle Medium (DMEM) containing 100 mM HEPES (pH 8.0) supplemented with 0.5 μM OsteoSense 680EX (PerkinElmer). After incubation at 25 °C for 60 minutes, 30 μl of the mixture was applied to a gel-filtration spin column (Bio-rad, molecular weight cut-off 40 kDa) and centrifuged at 1,000 g for 2 minutes. Fifty μl of the flow-through was diluted with the same volume of 2% SDS and 100 mM EDTA to avoid quenching, and subjected to the quantification of fluorescence using an infrared fluorescence scanner (Odyssey CLx, LI-COR, excitation at 685 nm, emission at 700 nm). The fluorescence intensity of OsteoSense was defined as the total CPP level. The same assay was repeated using the supernatant after the serum or plasma was centrifuged at 16,000 g for 2 hours to determine the low-density CPP (L-CPP) level. The high-density CPP (H-CPP) level was calculated by subtracting the L-CPP level from the total CPP level. Because the coefficient of variation in the gel filtration method is 0.022, we judged the H-CPP level below the detection limit when [H-CPP] over [total CPP] was less than 0.022. The CPP assay using the fetuin-A method was performed as previously described^7^.

### CPP synthesis

DMEM containing 2.0 mg/ml BSA and 0.5 mg/ml bovine fetuin-A (Sigma-Aldrich) were inoculated with phosphate buffer (pH 7.4) and CaCl_2_ to attain the final concentration of calcium and phosphate to be 2 mM and 3 mM, respectively. The mixture was incubated at 37 °C for 24 hours and used immediately.

### Pull-down of CPP from plasma

Alendronate, a primary amino-bisphosphonate, was immobilized on the surface of magnetic beads introduced with carboxylic acid groups (NHS Mag Sepharose, GE Healthcare) by amine coupling according to the manufacturer’s protocol. The magnetic beads surface-immobilized with alendronate (alendronate beads) or the magnetic beads treated with ethanolamine in the absence of alendronate (control beads) were added to plasma, incubated at 37 °C for 60 minutes, washed with DMEM for 5 times, and then eluted with saline containing 50 mM EDTA. The eluates were subjected to SDS-PAGE followed by silver staining. The ~50 kD band specifically pulled down with the alendronate beads was excised and identified as human fetuin-A by liquid chromatography coupled with tandem mass spectrometry (LC-MS/MS). In addition, the eluates were subjected to immunoblot analysis using antibodies against human fetuin-A (Cell Signaling) or annexin V (Abcam).

### Equipment and settings

The SDS-PAGE gel images were acquired using a digital camera (Canon IXY 170). The immunoblot images were acquired using ImageQuant LAS 4000 mini (GE Healthcare). Adjustment of the brightness and/or contrast was applied equally and linearly across the entire images using Adobe Photoshop CC (2015.5).

### OsteoSense binding to calcium-phosphate

Nanoparticles of amorphous calcium-phosphate (ACP), tricalcium phosphate (TCP), and hydroxyapatite (HA) purchased from Sigma-Aldrich were suspended in Krebs-Ringer-HEPES (KRH) with three different pH (6.8, 7.4, or 8.0) at the concentration of 0.1 mg/ml. The suspensions were freeze-thawed once or left untreated before inoculation of OsteoSense at the final concentration of 1 μM. After incubation at 25 °C for 60 minutes, the suspensions were centrifuged at 20,000 g for 30 minutes to precipitate the nanoparticles. The fluorescence intensity of OsteoSense in the supernatant was quantified to calculate the amount of OsteoSense bound to the nanoparticles.

### Measurement of particle size of CPP

The particle size distribution of CPP in plasma was determined by nanoparticle tracking analysis (NTA) using Nanosight NS300 (Nanosight, Amesbury, UK). In order to distinguish CPP from other colloidal particles in plasma, CPP were specifically labeled with FITC-alendronate, which was synthesized by amine coupling between FITC-carboxylic acid and the primary amine in alendronate. The FITC-alendronate was purified by HPLC and then added to plasma diluted with DMEM (1:4) at the final concentration of 1 μM. After incubation at 25 °C for 60 minutes, the mixture was subjected to NTA using the 488 nm laser and the 500 nm long pass filter.

### Preparation of human samples

In accordance with the protocol approved by the local ethics committee in Jichi Medical University and with the Declaration of Helsinki, total of 148 pre-dialysis CKD patients were recruited from outpatients at the Nephrology Department in Jichi Medical University Hospital between July 2014 and August 2017. Patients taking phosphate binders were not included in this study. Written informed consent was obtained from all participants. In each patient, serum and heparin plasma were separated from the whole blood by centrifugation at 1,200 g for 10 minutes within 60 minutes after blood sampling and aliquoted in several microcentrifuge tubes. Some tubes were designated as “fresh samples” and used immediately for the CPP assay. The other tubes were designated as “stored samples”, which were frozen and stored under the conditions described. Stored samples were used for measuring calcium (Ca), phosphate (P), magnesium (Mg), and creatinine (Cre), intact parathyroid hormone (iPTH), the active from of vitamin D (1,25(OH)_2_D_3_) as a part of the standard patients’ care. Fibroblast growth factor-23 (FGF23) was measured using ELISA (Kinos Laboratories, Inc.). Estimated glomerular filtration rate (eGFR) was calculated using the following formula: 194 × Age^−0.287^ × Cre^−1.094^ in males, 143.4 × Age^−0.287^ × Cre^−1.094^ in females.

### Statistics

Statistical analyses were performed using EZR (Jichi Medical University, Saitama, Japan), a graphical user interface for R (The R Foundation for Statistical Computing, Vienna, Austria)^14^ or Prism (version 6.0d, GraphPad Software, Inc.). Mann-Whitney U-test was used for evaluation of the difference in clinical parameters between H-CPP holders and non-holders. The Kolmogorov-Smirnov test was used to evaluate normality. Parameters that did not follow the normal distribution were normalized by logarithmic conversion and standardized for multivariate analysis. Pearson’s correlation coefficients (r) were calculated in univariate analysis. The covariates for multiple regression analysis were selected based on the following three criteria: 1) Parameters previously reported to correlate with serum CPP levels determined by the fetuin-A method (age, eGFR, iPTH, phosphate)^8^. 2) Parameters previously reported to affect propensity of secondary CPP formation i*n vitro* (calcium, phosphate, magnesium)^4^. 3) Endocrine factors that regulate mineral metabolism and thus potentially affect plasma CPP levels (FGF23, 1,25-dihydroxyvitamin D_3_).

## Electronic supplementary material

Supplementary information
