# Supplementary material for: Identification and quantification of plasma calciprotein particles with distinct physical properties in patients with chronic kidney disease
Source: Sci Rep. 2018 Jan 19;8:1256. doi: 10.1038/s41598-018-19677-4 (PMC5775250; doi:10.1038/s41598-018-19677-4)

Supplementary information

Identification and quantification of plasma calciprotein particles with distinct physical properties in patients with chronic kidney disease

Yutaka Miura[1], Yoshitaka Iwazu[1,2,3], Kazuhiro Shiizaki[1,3], Tetsu Akimoto[3], Kazuhiko Kotani[2,4], Masahiko Kurabayashi[5], Hiroshi Kurosu[1], and Makoto Kuro-o[1,6]

[1] Division of Anti-Ageing Medicine, Center for Molecular Medicine,

[2] Department of Clinical Laboratory Medicine,

[3] Division of Nephrology, Department of Internal Medicine,

[4] Division of Community and Family Medicine, Center for Community Medicine,

Jichi Medical University, Shimotsuke, Tochigi, Japan.

[5] Department of Cardiovascular Medicine, Gunma University Graduate School of Medicine, Maebashi, Gunma, Japan.

[6] AMED-CREST, Japan Agency for Medical Research and Development, Tokyo, Japan.

Correspondence to mkuroo@jichi.ac.jp

Full-length gels and blots

Figure 1c

Lanes 3 – 6 were shown in the Fig 1c. The other lanes were unrelated samples.

1 2 3 4 5 6 7 8 9 10 11 12 13 14 15

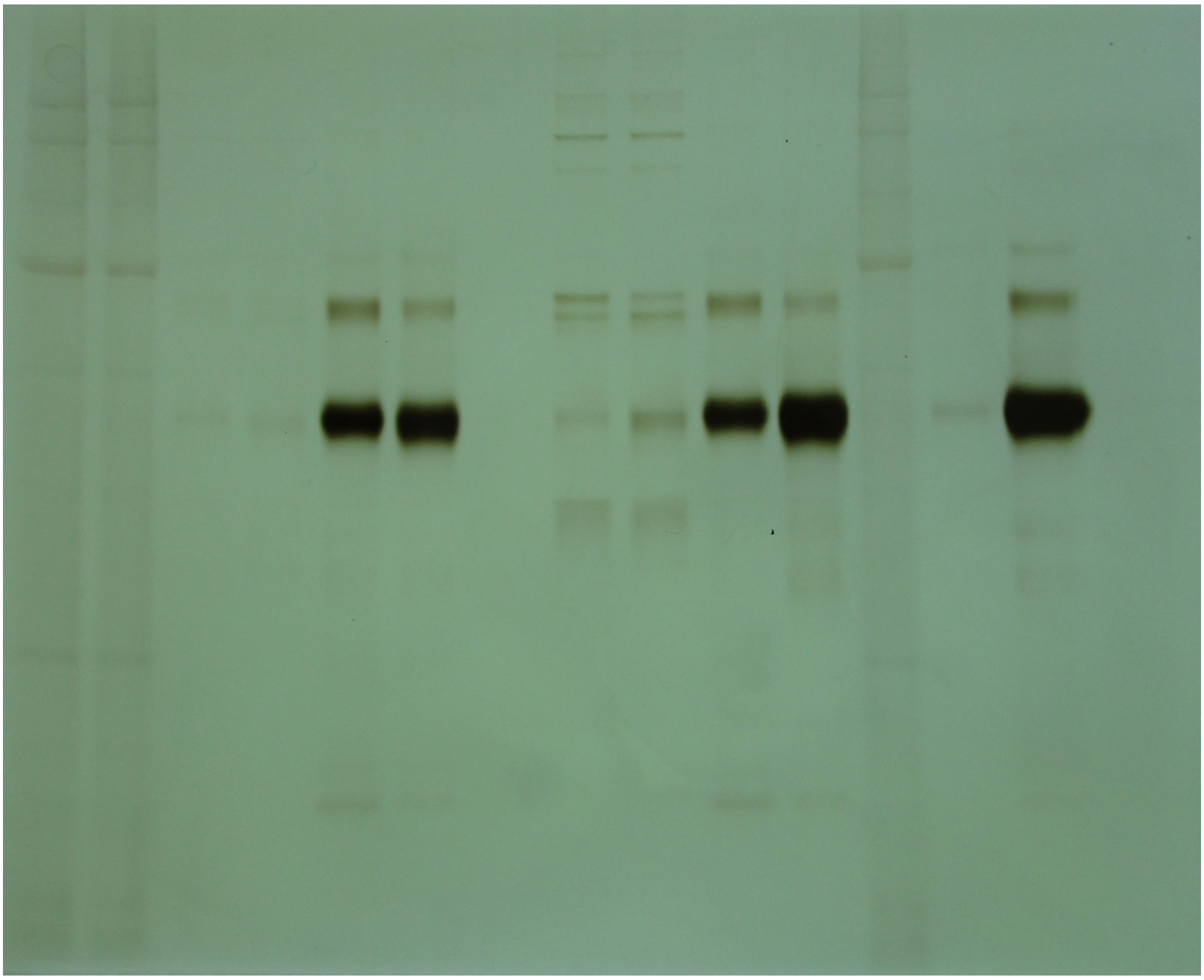

Figure 1d

Lanes 3 – 6 in Panel **C** were shown in the Fig 1d. The other lanes were unrelated samples. Because the brightness and contrast were linearly adjusted using Adobe Photoshop CC (2015.5), the original images with two different exposures were shown (Panel **A** anad **B**).

A

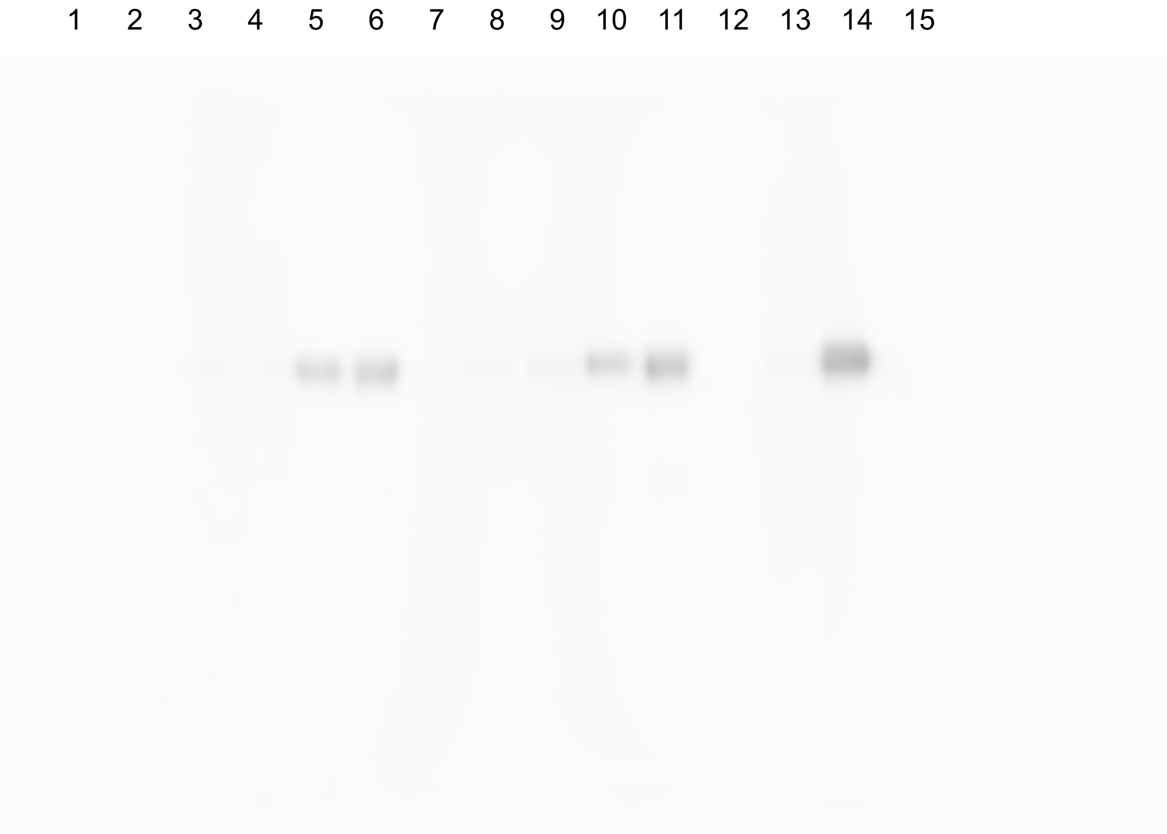

B

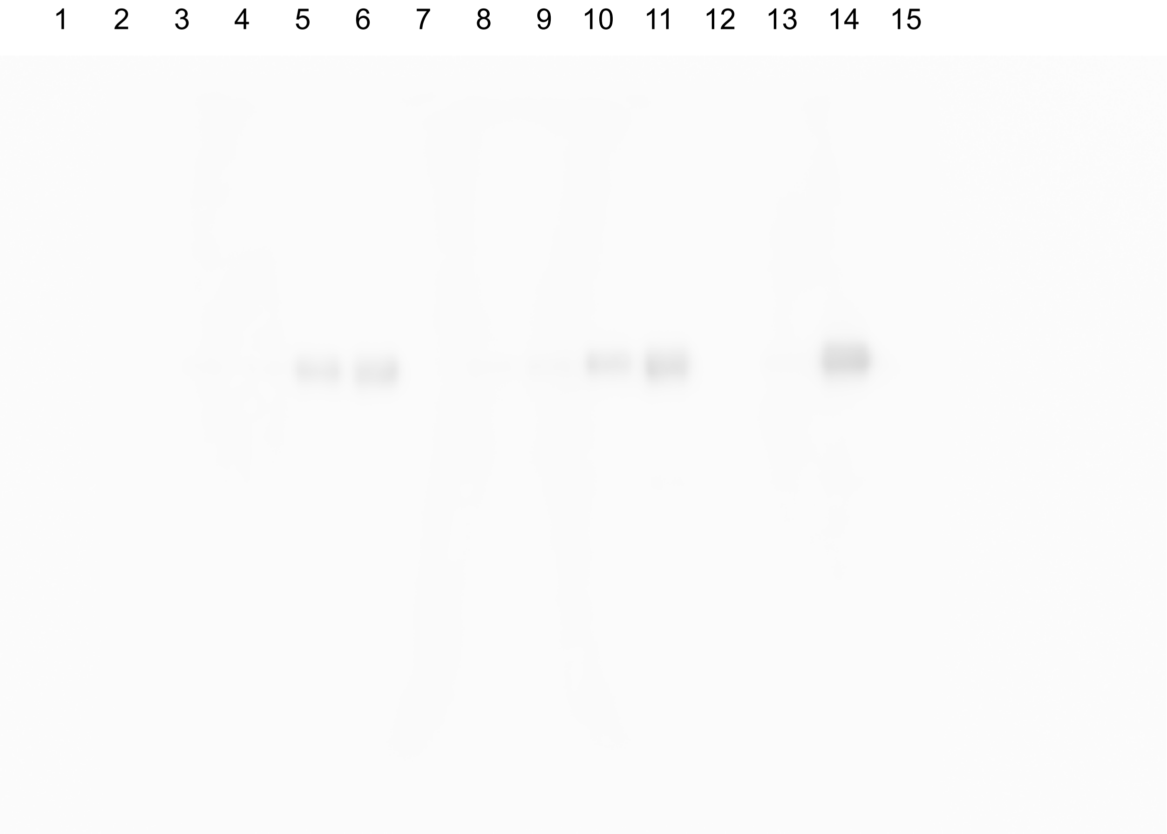

C

1 2 3 4 5 6 7 8 9 10 11 12 13 14 15

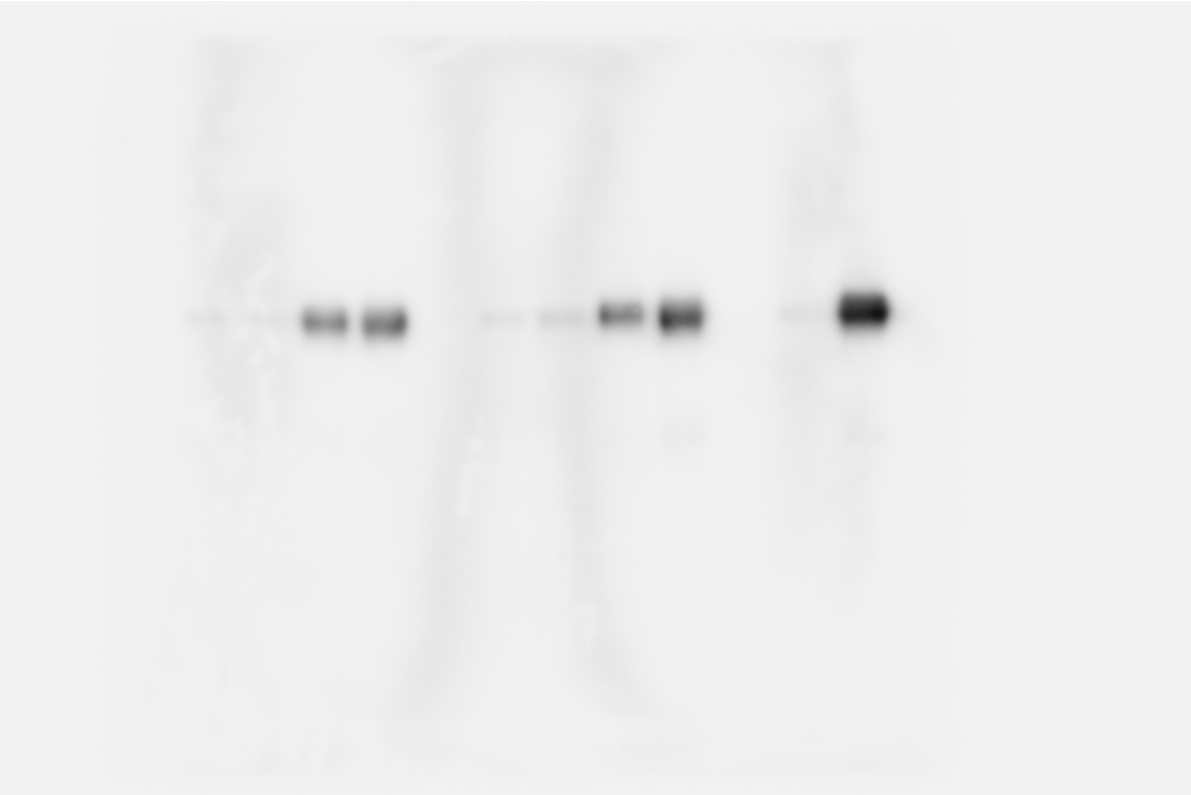

Figure 7c

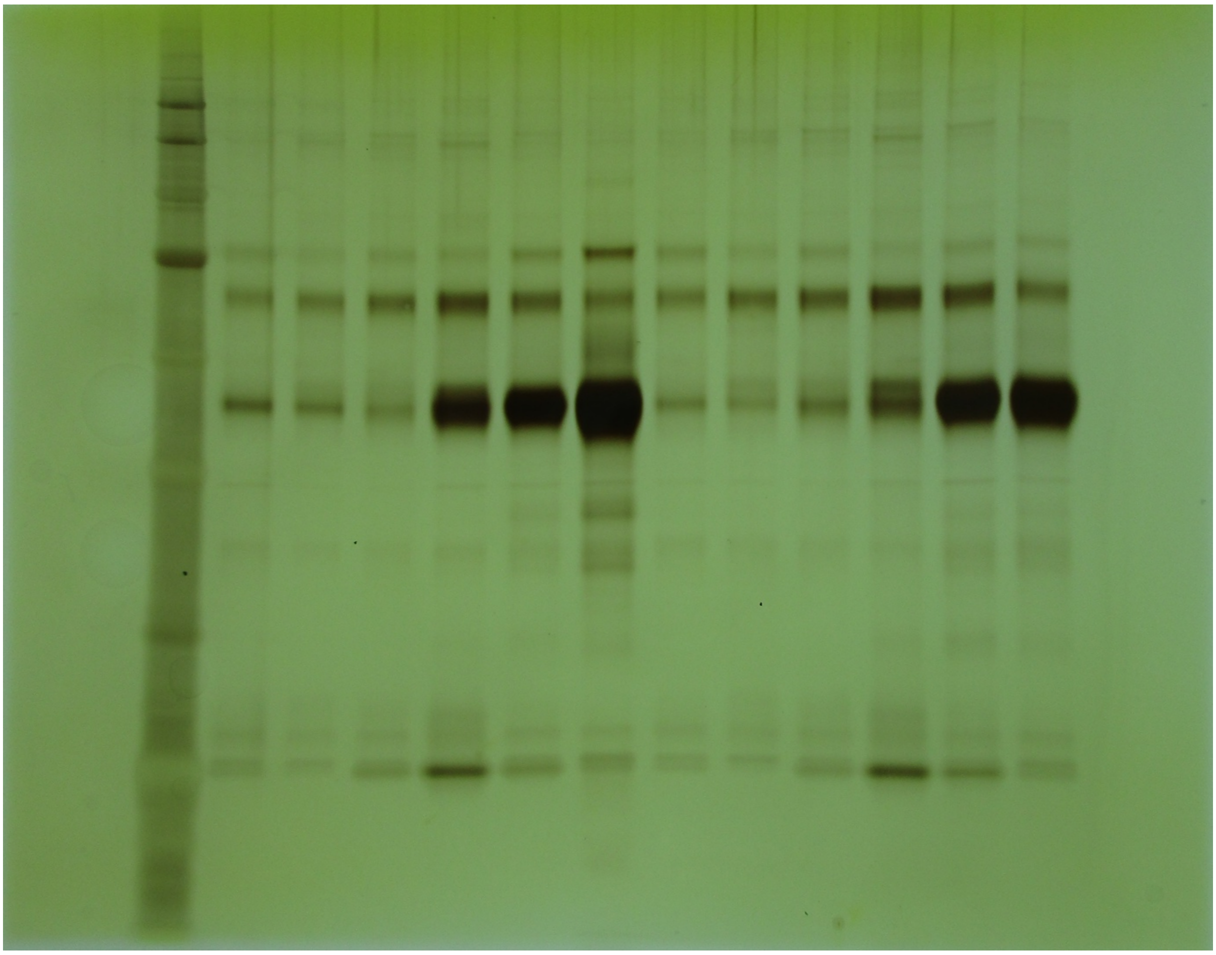

Supplement: Supplementary file 1 — Supplementary information [file 41598_2018_19677_MOESM1_ESM.pdf]
